# The Effect of Fermented Lingonberry Spray on Oral Health—A Pilot Study

**DOI:** 10.3390/dj13120568

**Published:** 2025-12-02

**Authors:** Hanna Lähteenmäki, Leo Pärnänen, Ismo T. Räisänen, Marjut Sakko, Pirjo Pärnänen, Timo Sorsa

**Affiliations:** Department of Oral and Maxillofacial Diseases, Head and Neck Canter, Helsinki University Hospital, University of Helsinki, 00014 Helsinki, Finland; leo.parnanen@helsinki.fi (L.P.); ismo.raisanen@helsinki.fi (I.T.R.); marjut.sakko@helsinki.fi (M.S.); pirjo.parnanen@helsinki.fi (P.P.); timo.sorsa@helsinki.fi (T.S.)

**Keywords:** *Vaccinium vitis idaea* L., phenolic compounds, fermented lingonberry juice, oral spray, saliva test, mouthrinse, aMMP-8 test

## Abstract

**Background/Objectives:** A fermented lingonberry juice spray (FLJ spray) was developed from lingonberry (*Vaccinium vitis idaea* L.) juice as a natural adjunct for oral health. It has a low sugar content and contains naturally occurring phenolic compounds to decrease oral microbial burden, inflammation, and dry mouth symptoms. This human intervention single-arm pilot study examined the oral effects of fermented lingonberry spray (FLJ spray) treatment, used for a period of 30 days. **Methods:** Eleven adult patients were recruited from a private dental clinic in Tampere, Finland. Traditional clinical oral examinations, periodontal status, and samples were collected at baseline, two weeks, and four weeks. Bleeding on probing (BOP), visible plaque index (VPI), and probing pocket depths (PPD) were examined, and active-matrix metalloproteinase-8 (aMMP-8) mouthrinse assays (cut off 20 ng/mL) were conducted. Additionally, stimulated and resting saliva, pH, and buffering capacity were assessed. A questionnaire assessing dry mouth symptoms was also recorded. **Results:** The effect of FLJ spray on clinical variables during the study period was analyzed by repeated measures ANOVA. Use of the Lingora^®^ spray reduced the assessed periodontal indices BOP (*p* < 0.05), VPI (*p* < 0.001), PPD ≥ 6 mm (*p* = 0.136), and aMMP-8 (*p* = 0.084). No adverse or contrary effects on the parameters were observed during the study. **Conclusions:** The use of FLJ spray improved periodontal status and reduced plaque burden and signs of inflammation, such as BOP and collagenolytic aMMP-8 levels. This pilot study suggests that FLJ spray is safe and appeared to be beneficial for use in addition to oral home care.

## 1. Introduction

Natural compounds have been increasingly studied to promote oral health. They offer an alternative to synthetic compounds commonly found from oral health products that may even pose a risk for antimicrobial resistance. Lingonberries (*Vaccinium vitis idaea* L.) are wild berries that grow in the northern hemisphere. They contain bioactive phenolic compounds that have antioxidative, anti-inflammatory, anti-proteolytic, and antimicrobial properties [1]. Lingonberry-specific cyanidins and other phenolic compounds downregulate in vitro inflammatory mediators, such as IL-6, TNF-α, IL-1β, MCP-1, COX2, and iNOS, upregulate extracellular matrix protein coding genes, resulting in tissue inflammation resolution, and inhibit activation of human latent pro-MMP-8 to its active aMMP-8 form in vitro and in vivo in the presence of inflammation. Fermented lingonberry mouthrinses have been clinically proven to be beneficial for periodontal disease patients by reducing microbial load (visible plaque), bleeding of the gums, and aMMP-8 levels [2,3] and increase salivary secretion [4]. Additional oral-hygiene-improving mouthrinse may be especially beneficial amongst the elderly with mouth-drying medications, or poor hand coordination, and even those with orthodontic fixed appliances that increase the risk of mucosal irritation, gingivitis, and dental decay [5,6]. Decreased salivary secretion causes the accumulation of plaque and increased risk of gingivitis, periodontitis, and dental decay.

Several different substances or drugs have been studied to prevent dry mouth, but no reliable results have been found for increasing saliva volume. Saliva is a protective gateway to the digestive system and has many different functions to maintain and manage oral health [7]. It participates in the mineralization and demineralization of tooth enamel and dentin. It also protects and lubricates the oral mucosa, has a buffering effect against acidity fluctuations after meals, improves taste, and initiates the digestive process. The decrease in saliva due to illness or age causes many different ailments for oral health [8,9,10,11]. The pH value of saliva is normally 6–7. Stimulated saliva refers to the volume of saliva produced when chewing paraffin, for instance, for five minutes. At this point, the flow rate of stimulated saliva is 1–3 mL/min, and the buffering capacity is 10–12. Eating or drinking causes changes in the pH and secretion rates of saliva, depending on the acidity, sugar content, and frequency of consumption of the beverage. Buffering occurs within 5–15 min [7,12].

In addition to mouthrinse and salivary testing, which enable easy and painless examination of oral health, it is possible to identify and quantify various inflammatory biomarkers from mouthrinse/oral fluid. A validated testing method that can be performed in a clinical setting is the aMMP-8 point-of-care (PoC) mouthrinse testing method with a cut-off of 20 ng/mL [13,14]. Previous studies have demonstrated the role of aMMP-8 in the manifestation of early periodontal diseases, as well as in promoting disease activity [15,16,17,18,19]. Research has shown that aMMP-8 levels in mouthrinse correlate well with and reflect clinical stage and grade of periodontitis and associated parameters [20,21]. The aMMP-8 testing method is highly accurate, and the numerical values obtained can be used to define oral health very precisely [19,22,23].

The objective of this intervention study was to evaluate the effects of the FLJ spray on bleeding on probing (BOP), visible plaque (VPI), and probing pocket depths (PPDs) and compare the effect of the spray formulation with lower dosing and application frequency compared to earlier studies with FLJ oral rinse. Effects on saliva secretion rates, saliva pH, and buffering capacity were assessed by using basic clinical saliva testing methods. Additionally, we used the aMMP-8 mouthrinse test method, which indexes and alarms early periodontal inflammatory conditions. Adult patients were recruited from a private dental clinic in Tampere, Finland, for the study. Our hypothesis was whether fermented lingonberry juice, administered as an oral spray four times a day after meals, could downregulate aMMP-8 levels and affect saliva flow and its buffering capacity without reducing pH, thereby alleviating symptoms of hyposalivation, dry mouth symptoms, and periodontitis without side effects.

## 2. Materials and Methods

### 2.1. Participants

Eleven adults (34–87 years, mean 62. M/F ratio 3/8) were recruited to a non-randomized pilot study from a dental hygienist office in Tampere. The study took place from September 2024 to April 2025. Healthy patients or patients with systemic diseases with good cognitive abilities were included in the study with or without xerostomia. Age, smoking habits, and presence of chronic diseases were recorded. Medical evaluations revealed underlying diseases such as heart disease and diabetes mellitus. Patients were excluded if they used other mouthwash products, were pregnant, had received antibiotic medications in the last 6 months, or if they had used CHX mouthwash in the last 6 months. No periodontal treatments were performed at the beginning of or during the trial. The patients used either a manual or an electric toothbrush twice daily. Patients were instructed to perform interdental cleaning with interdental brushes once a day using the same toothpaste (Oral-B Pro-Expert Professional Protection; Procter & Gamble, Cincinnati, OH, USA) and their normal home care tools for 30 days. The study was conducted in accordance with the Declaration of Helsinki and approved by the In-stitutional Review Board (or Ethics Committee) Regionala etikprövingsnämnden i Stockholm, ((EPN) 2016/1410-31; approve date: 25 November 2016). ClinicalTrials.gov Identifier: NCT07143344. The completed CONSORT 2010 checklist is provided as Supplementary Material Table S1.

### 2.2. Questionnaire

A questionnaire on subjective experiences regarding xerostomia was completed at the beginning and at the end of the study. The evaluation scale of the questions, along with a dichotomous response option (yes/no), and references to xerostomia were recorded, calculated, and a summary of scores was made for each patient. In the dry mouth sensation survey, the following questions were posed:

1. Does your mouth feel dry after eating?
2. Do you have difficulty swallowing?
3. Can you eat dry bread or cookies without drinking?
4. Do you feel that saliva production is low?
5. How often do you wake up at night due to feelings of dry mouth?

### 2.3. Tests

**Figure 1:**
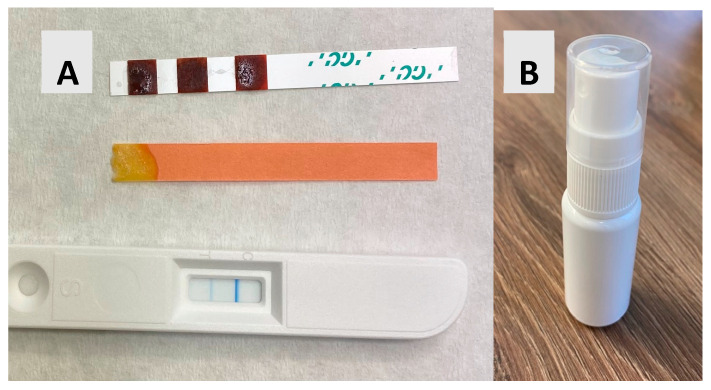
(**A**) Buffering capacity (very low), pH 6.2 and aMMP-8 test result (>20 ng/mL); (**B**) picture of an FLJ spray bottle.

Before the oral health examination, an aMMP-8 PoC (cut-off 20 ng/mL) chair-side lateral flow immunoassay (PerioSafe^®^ test, Dentognostics GmbH, Jena, Germany) was performed in conjunction with the digital reader (ORALyzer^®^, Dentognostics GmbH, Jena, Germany). aMMP-8 tests were carried out according to the manufacturer’s instructions (see Figure 1B).

The resting salivary flow rate and pH, stimulated salivary flow rate, and buffering capacity were measured and analyzed using the protocol specified by the manufacturer, Saliva-Check BUFFER test series (GC America Inc., Alsip, IL, USA), at baseline and 2 and 4 weeks later (see Figure 1B). Patients were instructed not to eat, drink, or brush their teeth for one hour prior to the study visit.

### 2.4. Clinical Examinations

After the saliva tests, clinical measurements were conducted. Indicators of oral health were utilized, such as the VPI, BOP, and PPD. Oral clinical investigation and diagnoses were performed by the same dentist at each timepoint. The depth of the periodontal pockets and BOP was recorded using a standard millimeter-graded American Eagle WHO probe (American Eagle Manufacturing Co., New Bern, NC, USA: AEEP23/WHOBX). The number of deepened periodontal pockets measuring 4–6 mm was recorded.

The determination of visible plaque and the measurement of gingival bleeding were conducted using six specified measurement points on the tooth, and each result obtained from the measurement points was recorded using values of VPI (scale ranging from VPI (0) = no plaque up to VPI (3) = plaque on all tooth surfaces) and BOP (yes/no).

### 2.5. Intervention

Participants used approximately ten sprays four times a day (approx. 5.5 mL/day) of FLJ spray (Lingora^®^ Oral Spray, Berries United Ltd., Helsinki, Finland) for a month. The participants were monitored for compliance by inquiring. The spray liquid contained phenolic compounds at 0.211% (*w*/*v*) and had a pH of 2.77 (see Figure 1) FLJ spray was manufactured using a patented method, similarly to the one used to create FLJ oral mouthrinse [24]. Patients used fluoride-containing toothpaste for oral hygiene throughout the study. This study has received ethical approval, and informed consent was obtained from all participants. This study complies with the STROBE guidelines protocol.

### 2.6. Statistics

The overall differences between the timepoints (t1, t2, t3) on the levels of each salivary parameter were determined with the repeated measures ANOVA, and pairwise comparisons from the repeated measures ANOVA were adjusted for multiple comparisons using Bonferroni’s post hoc test. Mauchly’s test was used to assess the assumption of sphericity in repeated measures ANOVA. For aMMP-8 and PPD, the assumption was violated and based on the epsilon value, which was less than 0.75; the Greenhouse–Geisser correction was applied to increase the robustness of the ANOVA. Furthermore, there were no missing data. There was a potential outlier in one patient’s BOP levels in the third/final timepoint (t1 = 15%…t2 = 10%…t3 = 42%), but BOP reduction during the study stayed significant regardless of removing the outlier from the repeated measures ANOVA. The patient had no other outliers in his/her other clinical parameters. Another assumption of repeated measures ANOVA, the normality of variables, was assessed by the Shapiro–Wilk test and QQ-plot. The normality of BOP in t3, VPI in t3, and aMMP-8 in t1, t2, t3 could not be confirmed. However, for BOP, VPI, PPD, and aMMP-8, the repeated measures ANOVA results agreed with the non-parametric Friedman test result. A two-tailed *p*-value below 0.05 was considered statistically significant. Statistical analyses were made using the SPSS version 29.0.2.0 (IBM SPSS Statistics for Windows, IBM Corp., Armonk, NY, USA).

This was a pilot study, and analyses were performed without a priori power calculation. Some of the results in this study reached the level of significance, indicating enough power in this study. Before this study, there was not enough information about the effect of FLJ spray to estimate the effect sizes. The present results can be utilized in power analysis to calculate the sample size better for future studies to further investigate this subject and confirm the results of this study.

## 3. Results

### 3.1. Demographics

A total of 11 of the recruited participants used the oral spray as instructed (4 times per day), and they were included in the analyses. The final sample number and the ages, gender, and systemic diseases of the participants are shown in Table 1. All participants attended all three research visits.

### 3.2. Saliva/Xerostomia

There were no observed significant differences in the flow rates of resting or stimulated saliva, pH saliva values, nor buffering capacity during the period. The results of the subjective questionnaire on xerostomia indicated that subjective symptoms of dry mouth did not change during the FLJ spray period. None of the individuals reported side effects, and they considered the spray to be easy to use (see Table 2).

**Figure 2:**
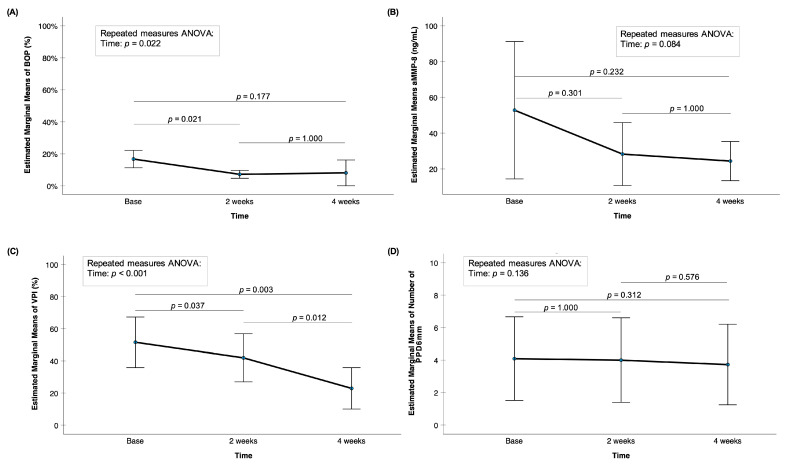
Mean levels of (**A**) BOP (%), (**B**) aMMP-8 (ng/mL), (**C**) VPI (%), and (**D**) the number of PPD ≥ 6 mm with 95% confidence interval bars for timepoints of base, 2 weeks, and 4 weeks of FLJ spray therapy. *p*-value of “Repeated measures ANOVA: Time” indicates if there was an overall significant difference between the means at the different timepoints of base, 2 weeks, and 4 weeks of FLJ spray therapy. All pairwise comparisons from repeated measures ANOVA were adjusted for multiple comparisons (the Bonferroni post hoc test) and are marked in the figure.

FLJ spray significantly reduced BOP (*p* = <0.05) and VPI (*p* < 0.001); marginal means of PPD ≥ 6 mm (*p* = 0.136) decreased slightly but not significantly. Also, aMMP-8 levels decreased, especially after the first two weeks of use, but not significantly (*p* = 0.084). aMMP-8 levels also stayed for the following week. Thus, no significant reverse increases in the clinical parameters and active MMP-8 mouthrinse levels were noted during the study (see Figure 2).

## 4. Discussion

### 4.1. Comparison with Previous Studies

Previous studies on the use of FLJ as mouthwash [2,3,24] have been promising to show antibacterial, anticandidal, and anti-inflammatory effects. A longer (6 months) FLJ mouthrinse period has been verified [4] to increase salivary secretion rates and buffering capacity and may be beneficial to relieve dry mouth symptoms more efficiently. The current study utilized a short duration of one month, during which individuals did not notice significant differences in their dry mouth symptoms. However, feedback discussions revealed that individuals experienced relief from the sensation of dry mouth, as the oral spray was used throughout the day.

### 4.2. Explanation of Biological Mechanisms

Several in vitro studies have demonstrated that the phenolic compounds in lingonberries exhibit antibacterial [25,26,27], anti-viral [28], anti-proteolytic [29], and anti-inflammatory effects [30]. There are studies which indicate that consuming lingonberries improves systemic health, immune responses, and metabolic functions and also decreases intestinal inflammation [31,32,33]. The effects of FLJ on the health of connective tissues [18,19,30], oral yeast infections [2,24], and peri-implant tissues [3] have been studied, yielding promising clinical and oral fluid biomarker outcomes. A previous study revealed that FLJ can inhibit the activation of pro-MMP-8 caused by a cell wall proteinase fraction of *Candida glabrata* [29]. For this reason, we included aMMP-8 testing in this study, which demonstrated that FLJ can reduce low-grade oral inflammation after just one month of use.

### 4.3. Main Findings and Clinical Relevance

The significant reduction in BOP and VPI help maintain oral home care. Saliva pH increased slightly, and this confirms and further extends the results found by Pärnänen et al. Dent J 2022 [19], that FLJ in spray form may not cause damage to teeth related to acidity. Also, aMMP-8 mouthrinse levels decreased close to 20 ng/mL, a threshold value discriminating periodontal health and disease [20,34], indicating a reduction in the proteolytic tissue destruction burden. Furthermore, after initial reductions in clinical periodontal indices and aMMP-8 mouthrinse biomarker levels during the study period, there was no detectable reverse increases in clinical and biomarker parameters. Overall, these results suggest that the FLJ spray therapeutic intervention, as performed in this study, may support the maintenance phase of periodontal treatments and justify further clinical studies.

### 4.4. Limitations and Future Studies

Regarding the limitations of this exploratory pilot study, which needs to be confirmed and further extended by larger placebo-controlled randomized studies, a longer-duration FLJ spray-use period and including a control group may corroborate these results. In the present pilot study, analyses were performed without an a priori power calculation because there was not enough information about the effect of FLJ spray to estimate the effect sizes. Nevertheless, some of the results in this study reached the level of significance, indicating enough power in this study. The present results can be utilized in power analysis for future studies to calculate the required sample size more accurately to further investigate this subject and confirm the results of this study.

## 5. Conclusions

FLJ is safe to use, as there are no known interactions with other medications or side effects. The FLJ spray discussed in this study is very healthy, easy to use, and practical for the patient. Due to the fermentation process, its natural sugar content is very low, and the recommended amount has positive effects on oral health in gum disease and those suffering from dry mouth. Considering the significant role played by saliva as a defense mechanism and the ability of natural substances such as fermented lingonberry juice to aid in oral defense, larger randomized placebo-controlled studies are required to validate our preliminary findings.

## Figures and Tables

**Table 1:** Patient characteristics.

Age (mean standard deviation)	62.81 ± 14
Sex (female/male %)	27.3 /72.7
Smoking /Snuff (Yes%)	18
Heart diseases (%)	27.2
Asthma (%)	18
Rheumatism (%)	18
Diabetes (%)	9

**Table 2:** Frequency of participants in each classification of variables during the study.

Saliva Samplings	Timepoint 1	Timepoint 2	Timepoint 3
Resting saliva flow (N)			
Low	6	6	6
Normal	5	5	5
Stimulated saliva (N)			
Extremely low (<3.5 mL/min)	1	1	1
Low (3.5–5.0 mL/min)	3	3	2
Normal (>5.0 mL/min)	7	7	7
Resting saliva pH (N)			
Highly acidic (5–5.8)	0	0	0
Moderately acidic (6–6.6)	2	1	1
Healthy saliva (6.8–7.8)	9	10	10
Buffering capacity (N)			
Very low (0–5)	4	4	4
Low (6–9)	1	0	0
Normal (10–12)	6	7	7

## Data Availability

Data supporting the reported results can be obtained from the authors on request.

## Supplementary Materials

The following supporting information can be downloaded at: https://www.mdpi.com/article/10.3390/dj13120568/s1, Table S1: CONSORT 2010 checklist of information to include when reporting a randomised trial.

## References

[1] Pärnänen P., Lähteenmäki H., Tervahartiala T., Räisänen I.T., Sorsa T. (2021). Lingonberries—General and Oral Effects on the Microbiome and Inflammation. Nutrients.

[2] Pärnänen P., Lomu S., Räisänen I.T., Tervahartiala T., Sorsa T. (2023). Antimicrobial and Anti-Inflammatory Oral Effects of Fermented Lingonberry Juice-A One-Year Prospective Human Intervention Study. Eur J Dent..

[3] Lähteenmäki H., Tervahartiala T., Räisänen I.T., Pärnänen P., Sorsa T. (2022). Fermented lingonberry juice’s effects on active MMP-8 (aMMP-8), bleeding on probing (BOP), and visible plaque index (VPI) in dental implants—A clinical pilot mouthwash study. Clin. Exp. Den.T Res..

[4] Pärnänen P., Lomu S., Räisänen I.T., Tervahartiala T., Sorsa T. (2022). Effects of Fermented Lingonberry Juice Mouthwash on Salivary Parameters—A One-Year Prospective Human Intervention Study. Dent. J..

[5] Manuelli M., Marcolina M., Nardi N., Bertossi D., De Santis D., Ricciardi G., Luciano U., Nocini R., Mainardi A., Lissoni A. (2019). Oral mucosal complications in orthodontic treatment. Minerva Stomatal..

[6] Lucchese A., Carinci F., Brunelli G., Monguzzi R. (2011). An in vitro study of resistance to corrosion in brazed and laser-welded orthodontic appliances. Eur. J. Inflamm..

[7] Schipper R.G., Silletti E., Vingerhoeds M.H. (2007). Saliva as research material: Biochemical, physicochemical and practical aspects. Arch. Oral Biol..

[8] Lenander-Lumikari M., Loimaranta V. (2000). Saliva and dental caries. Adv. Dent. Res..

[9] Martina E., Campanati A., Diotallevi F., Offidani A. (2020). Saliva and Oral Diseases. J. Clin. Med..

[10] Buzalaf M.A., Hannas A.R., Kato M.T. (2012). Saliva and dental erosion. J. Appl. Oral Sci..

[11] Hans R., Thomas S., Garla B., Dagli R.J., Hans M.K. (2016). Effect of Various Sugary Beverages on Salivary pH, Flow Rate, and Oral Clearance Rate amongst Adults. Scientifica.

[12] Zhang C.Z., Cheng X.Q., Li J.Y., Zhang P., Yi P., Xu X., Zhou X.D. (2016). Saliva in the diagnosis of diseases. Int. J. Oral Sci..

[13] Al Habobe H., Haverkort E.B., Nazmi K., Van Splunter A.P., Pieters R.H.H., Bikker F.J. (2024). The impact of saliva collection methods on measured salivary biomarker levels. Clin. Chim Acta.

[14] Sorsa T., Tjäderhane L., Salo T. (2004). Matrix metalloproteinases (MMPs) in oral diseases. Oral Dis..

[15] Sorsa T., Gursoy U.K., Nwhator S., Hernandez M., Tervahartiala T., Leppilahti J., Gursoy M., Könönen E., Emingil G., Pussinen P.J. (2016). Analysis of matrix metalloproteinases, especially MMP-8, in gingival creviclular fluid, mouthrinse and saliva for monitoring periodontal diseases. Periodontology 2000.

[16] Sorsa T., Gieselmann D., Arweiler N.B., Hernández M. (2017). A quantitative point-of-care test for periodontal and dental peri-implant diseases. Nat. Rev. Dis. Primers..

[17] Räisänen I.T., Heikkinen A.M., Siren E., Tervahartiala T., Gieselmann D.R., van der Schoor G.J., van der Schoor P., Sorsa T. (2018). Point-of-Care/Chairside aMMP-8 Analytics of Periodontal Diseases’ Activity and Episodic Progression. Diagnostics.

[18] Alassiri S., Parnanen P., Rathnayake N., Johannsen G., Heikkinen A.M., Lazzara R., van der Schoor P., van der Schoor J.G., Tervahartiala T., Gieselmann D. (2018). The Ability of Quantitative, Specific, and Sensitive Point-of-Care/Chair-Side Oral Fluid Immunotests for aMMP-8 to Detect Periodontal and Peri-Implant Diseases. Dis. Markers.

[19] Al-Majid A., Alassiri S., Rathnayake N., Tervahartiala T., Gieselmann D.R., Sorsa T. (2018). Matrix Metalloproteinase-8 as an Inflammatory and Prevention Biomarker in Periodontal and Peri-Implant Diseases. Int. J. Dent..

[20] Sorsa T., Alassiri S., Grigoriadis A., Räisänen I.T., Pärnänen P., Nwhator S.O., Gieselmann D.R., Sakellari D. (2020). Active MMP-8 (aMMP-8) as a Grading and Staging Biomarker in the Periodontitis Classification. Diagnostics.

[21] Lähteenmäki H., Umeizudike K.A., Heikkinen A.M., Räisänen I.T., Rathnayake N., Johannsen G., Tervahartiala T., Nwhator S.O., Sorsa T. (2020). aMMP-8 Point-of-Care/Chairside Oral Fluid Technology as a Rapid, Non-Invasive Tool for Periodontitis and Peri-Implantitis Screening in a Medical Care Setting. Diagnostics.

[22] Sorsa T., Bacigalupo J., Könönen M., Pärnänen P., Räisänen I.T. (2020). Host-Modulation Therapy and Chair-Side Diagnostics in the Treatment of Peri-Implantitis. Biosensors.

[23] Verhulst M.J.L., Teeuw W.J., Bizzarro S., Muris J., Su N., Nicu E.A., Nazmi K., Bikker F.J., Loos B.G. (2019). A rapid, non-invasive tool for periodontitis screening in a medical care setting. BMC Oral Health.

[24] Pärnänen P., Nikula-Ijäs P., Sorsa T. (2019). Antimicrobial and Anti-inflammatory Lingonberry Mouthwash—A Clinical Pilot Study in the Oral Cavity. Microorganisms.

[25] Nohynek L.J., Alakomi H.L., Kähkönen M.P., Heinonen M., Helander I.M., Oksman-Caldentey K.M., Puupponen-Pimiä R.H. (2006). Berry phenolics: Antimicrobial properties and mechanisms of action against severe human pathogens. Nutr. Cancer.

[26] Heinonen M. (2007). Antioxidant activity and antimicrobial effect of berry phenolics—A Finnish perspective. Mol. Nutr. Food Res..

[27] Riihinen K.R., Ou Z.M., Gödecke T., Lankin D.C., Pauli G.F., Wu C.D. (2014). The antibiofilm activity of lingonberry flavonoids against oral pathogens is a case connected to residual complexity. Fitoterapia.

[28] Nikolaeva-Glomb L., Mukova L., Nikolova N., Badjakov I., Dincheva I., Kondakova V., Doumanova L., Galabov A.S. (2014). In vitro antiviral activity of a series of wild berry fruit extracts against representatives of Picorna-, Orthomyxo- and Paramyxoviridae. Nat. Prod. Commun..

[29] Pärnänen P., Sorsa T., Tervahartiala T., Nikula-Ijäs P. (2020). Isolation, characterization and regulation of moonlighting proteases from Candida glabrata cell wall. Microb. Pathog..

[30] Kylli P., Nohynek L., Puupponen-Pimiä R., Westerlund-Wikström B., Leppänen T., Welling J., Moilanen E., Heinonen M. (2011). Lingonberry (Vaccinium vitis-idaea) and European cranberry (Vaccinium microcarpon) proanthocyanidins: Isolation, identification, and bioactivities. J. Agric. Food Chem..

[31] Kivimäki A.S., Ehlers P.I., Siltari A., Turpeinen A.M., Vapaatalo H., Korpela R. (2012). Lingonberry, cranberry and blackcurrant juices affect mRNA expressions of inflammatory and atherothrombotic markers of SHR in a long-term treatment. J. Funct. Foods.

[32] Kivimäki A.S., Siltari A., Ehlers P.I., Korpela R., Vapaatalo H. (2014). Lingonberry juice negates the effects of a high salt diet on vascular function and low-grade inflammation. J. Funct. Foods.

[33] Marungruang N., Kovalenko T., Osadchenko I., Voss U., Huang F., Burleigh S., Ushakova G., Skibo G., Nyman M., Prykhodko O. (2020). Lingonberries and their two separated fractions differently alter the gut microbiota, improve metabolic functions, reduce gut inflammatory properties, and improve brain function in ApoE−/− mice fed high-fat diet. Nutr. Neurosci..

[34] Penttala M., Sorsa T., Thomas J.T., Grigoriadis A., Sakellari D., Sahni V., Gupta S., Pärnänen P., Pätilä T., Räisänen I.T. (2025). Determination of the Stage of Periodontitis with 20 ng/mL Cut-Off aMMP-8 Mouth Rinse Test and Polynomial Functions in a Mobile Application. Diagnostics.

